# Joining Alumina to Titanium Alloys Using Ag-Cu Sputter-Coated Ti Brazing Filler

**DOI:** 10.3390/ma13214802

**Published:** 2020-10-28

**Authors:** Omid Emadinia, Aníbal Guedes, Carlos José Tavares, Sónia Simões

**Affiliations:** 1 Department of Metallurgical and Materials Engineering, University of Porto, Rua Dr. Roberto Frias, 4200-465 Porto, Portugal; omid.emadinia@fe.up.pt; 2 LAETA/INEGI—Institute of Science and Innovation in Mechanical and Industrial Engineering, Rua. Dr. Roberto Frias, 4200-465 Porto, Portugal; 3 Department of Mechanical Engineering, CMEMS-UMinho, University of Minho, Azurém, 4800-058 Guimarães, Portugal; aguedes@dem.uminho.pt; 4 Centre of Physics, University of Minho, Azurém, 4800-058 Guimarães, Portugal; ctavares@fisica.uminho.pt

**Keywords:** alumina, γ-TiAl alloy, Ti6Al4V, brazing, interface, microstructure, microhardness

## Abstract

The joining of alumina (Al_2_O_3_) to γ-TiAl and Ti6Al4V alloys, using Ag-Cu sputter-coated Ti brazing filler foil, was investigated. Brazing experiments were performed at 980 °C for 30 min in vacuum. The microstructure and chemical composition of the brazed interfaces were analyzed by scanning electron microscopy and by energy dispersive X-ray spectroscopy, respectively. A microstructural characterization of joints revealed that sound multilayered interfaces were produced using this novel brazing filler. Both interfaces are composed mainly of α-Ti, along with Ti_2_(Ag,Cu) and TiAg intermetallics. In the case of the brazing of γ-TiAl alloys, α_2_-Ti_3_Al and γ-TiAl intermetallics are also detected at the interface. Bonding to Al_2_O_3_ is promoted by the formation of a quite hard Ti-rich layer, which may reach a hardness up to 1872 HV 0.01 and is possibly composed of a mixture of α-Ti and Ti oxides. Hardness distribution maps indicate that no segregation of either soft or brittle phases occurs at the central regions of the interfaces or near the base Ti alloys. In addition, a smooth hardness transition was established between the interface of Al_2_O_3_ to either γ-TiAl or Ti6Al4V alloys.

## 1. Introduction

High wear resistance, excellent thermal stability, or even thermal and electrical conductivity are attractive properties of advanced ceramics such as Al_2_O_3_ and zirconia. These ceramics are extensively applied in electronics, aerospace, nuclear and transportation industries [1]. However, the application of ceramics is limited for large production and complex shaped components due to inherit brittleness, hardness, and cost [2,3].

Titanium alloys are particularly attractive due to them having a low density, high specific strength and excellent corrosion resistance. However, poor high-temperature oxidation resistance limits the operating temperature of conventional Ti alloys to around 500 °C. In spite of this shortcoming, there is a growing interest in the development and joining of Ti-based alloys such as γ-TiAl, TiNi and Ti6Al4V [4,5].

The joining between Ti alloys and advanced ceramics can be a good option to overcome the limitations of Ti alloys at high temperatures. Dissimilar bonding allows the combination of these materials’ properties, extending their potential applications for the automotive and aerospace industries. The use of a ceramic part on a specific section of a complex metallic structure may grant an adequate performance at higher service temperatures. Nevertheless, dissimilar joining is particularly challenging. Indeed, due to the inherent differences in physical, thermal and chemical properties between metals and ceramics, it is quite difficult to produce adequate metal/ceramic joints. The application of ultrasonic bonding [6,7], electrical discharge pulse welding [8,9], solid state diffusion bonding [10,11], brazing [12,13], and laser lap joining [14] were reported in the literature for the joining of dissimilar materials. Moreover, some authors have prepared comprehensive reviews covering this field [15,16]. Brazing is widely used for the dissimilar bonding between advanced ceramics and Ti alloys. A major advantage of brazing, in comparison to other joining techniques, is related to the ease of producing interfaces that effectively buffer the residual stresses that arise from the mismatch between the coefficients of thermal expansion (CTEs) of ceramics and Ti alloys [17,18]. The commercial active brazing fillers used are often based on the Ag-Cu eutectic, with small additions of Ti which acts as an active element. However, joints resulting from brazing with Ag-based fillers usually present extensive segregation of (Ag) at the center of the interface [19], which limits the service temperature of joints to around 350 °C [20].

The success of brazing depends on having the appropriate heating apparatus, adequate temperature, and proper composition of brazing alloy. These parameters influence the microstructure of the brazed interface, which in turn plays a crucial role in the mechanical integrity and performance of joints. Paiva et al. [21] studied the influence of brazing temperature (850, 900 and 950 °C) on the microstructure and the shear strength of Al_2_O_3_/Ti joints brazed with an Ag-26.5Cu-3Ti (in wt.%) filler alloy. Though a brazing temperature increase resulted in the reduction in (Ag) and the increase in Ti at the interface, a decrease in the shear strength was observed and correlated with the formation of pores and cracks. A maximum shear strength was obtained for the joint processed at 850 °C for 40 min. Wu et al. [22] used a (Ag-28Cu)-3Ti foil of 190 µm thick to braze Al_2_O_3_ to Ti6Al4V alloy, and evaluated the influence of brazing temperature. The authors remarked the formation of complex oxide layers (Cu_2_Ti_4_O/Cu_4_Ti_3_O) at the vicinity of Al_2_O_3_ for joints brazed at 800 and 850 °C. By increasing the brazing temperature to 900 °C, this oxide transformed to another complex oxide type (Cu_3_Ti_5_O + CuAl_2_O_4_). It was reported to be (Ag) at all interfaces.

The brazing of an Al_2_O_3_ to Ti6Al4V alloy was also reported using filler alloys composed of TiB_2_ additive ball milled with Cu-Ti powder [23], the same additive milled with Cu powder [24], a commercial Ag-Cu-Ti powder mixed with B powder [25], and Ag-Cu eutectic powders mixed with different contents of B [3]. The common aspect of these studies involved the use of B for in situ formation of TiB whiskers in order to minimize the CTE between the base materials. It was also reported in these studies that the joint strength decreased when proper values of either brazing temperature or holding time were exceeded.

Cao et al. [26] evaluated the influence of an intermediate Cu layer (in 10, 40 and 120 µm) on a sandwiched eutectic Ag-Cu filler for brazing an Al_2_O_3_ to Ti6Al4V alloy. Brazing experiments were conducted between 800 and 950 °C, with holding times between 5 and 60 min; the maximum shear strength was obtained for joining at 825 °C for 10 min.

Regarding the brazing of the Al_2_O_3_ to γ-TiAl alloy, the use of active Ag-Cu-Ti powder brazing mixtures with TiH_2_ and B additives [17,27] and W additives [28] was reported. In these studies, the formation of complex oxides was observed at the Al_2_O_3_ side of the joints. The nature of these compounds was reported to be influenced by brazing temperature and the type of additive used. The use of a 50-µm foil of Ag-21Cu-4.5Ti (in wt.%) for brazing between 860 and 940 °C for 1 to 15 min [29] was reported to induce the formation of (Ag) and (Cu) at the interface complex oxide ((Cu,Al)_3_TiO_3_), which was observed in the vicinity of Al_2_O_3_. The maximum shear strength was obtained for joints processed at 900 °C, with a dwelling stage of 5 min.

A novel active filler, consisting of a Ti foil sputter-coated with Ag and Cu films, has been reported to be effective for brazing a γ-TiAl alloy to Hastelloy [30], and for producing similar γ-TiAl alloy joints [31]. γ-TiAl alloy/Hastelloy joints were processed at 900, 950 and 980 °C, but interfaces free of pores, cracks and without unbonded zones were only achieved after brazing at 980°C. The multilayered interfaces consisted mainly of Ti-Al and Ti-Ni-Al intermetallics close to the γ-TiAl alloy, and of Ti-rich, Ti-Ni, and Cr-Ni-Mo-rich phases near Hastelloy. The hardness of the interface, ranging from around 300 to 1100 HV0.01, was higher than both base materials, but no segregation of either (Ag) or coarse intermetallic particles was observed. For similar γ-TiAl alloy joints, brazing was successfully achieved at 950 and 980 °C. The interfaces consisted of a large central region composed essentially of α-Ti but also of Ti-Al and Ti-Ag compounds. Near the base of the γ-TiAl alloy, the interface consists of thin layers, mainly composed of Ti-Al intermetallics. In comparison to commercial Ag-Cu eutectic-based brazing alloys, the use of this Ag-Cu sputter-coated Ti brazing filler prevented the formation of either large amounts of (Ag) and hard phases at the interfaces, potentially enhancing the operating temperature of joints and eliminating the need of post-brazing heat treatments [20]. The reported results are promising for the prospective use of this filler for brazing other systems of dissimilar materials such as ceramics and metals.

In this context, the aim of the present study is to evaluate the use of this novel Ag-Cu sputter-coated Ti brazing filler in the joining of Al_2_O_3_ to γ-TiAl and to Ti6Al4V alloys. The interfacial microstructural and chemical features of joints were analyzed by scanning electron microscopy (SEM) and by energy dispersive X-ray spectroscopy (EDS). The mechanical properties of the brazed interfaces were evaluated by the Vickers microhardness tests and a possible reaction mechanism promoting bonding is discussed.

## 2. Materials and Methods

The base materials used in this study were Al_2_O_3_, with a relative density of 95.7%, a γ-TiAl alloy (Ti-45Al-5Nb (at. %)), with a duplex microstructure (a mixture of γ-TiAl grains and of γ + α_2_ lamellar grains), and a Ti6Al4V α-β alloy. The base materials were wet ground with SiC paper to a 1200 mesh finish.

The production conditions as well as the microstructural characterization of the Ag-Cu sputter-coated Ti brazing filler were previously described [30,31]. The filler is composed of a Ti foil coated in both sides with a Ag film (20 µm) followed by a Cu film (5 µm); the total thickness of the filler is 150 µm. Prior to brazing, all materials were degreased in acetone with ultrasonic agitation and dried in air. The brazing experiments were conducted by a resistant furnace with a horizontal alumina tube, at 980 °C for 30 min in vacuum (better than 10^−4^ mbar) with heating and cooling rates set to 5 °C/min. This temperature was selected based on the better results in the brazing of similar and dissimilar γ-TiAl alloys using the same filler alloy [30,31].

Joints for microstructural characterization were prepared using conventional metallographic techniques. The microstructure of the interfaces was characterized by optical microscopy (OM) using for DM4000 equipment (Leica Microsystems, Wetzlar, Germany) and by SEM using a FEI QUANTA 400 FEG equipment (Hillsboro, OR, USA). Chemical analysis of the interfaces was performed by the EDS technique (Oxford Instrument, Oxfordshire, UK). SEM/EDS analyses were performed at an accelerating voltage of 15 keV for mapping and for local analysis using the standardless quantification method.

The mechanical behavior of the of brazed interfaces was assessed by microhardness measurements on the polished interfaces. Vickers microhardness tests were performed with a 98-mN load (HV0.01) using Duramin-1 Struers equipment (Ballerup, Denmark). The hardness maps were obtained by indentation matrices up to 10 rows per 14 columns.

## 3. Results and Discussion

The success of using the Ag-Cu sputter-coated Ti brazing filler in the joining of Al_2_O_3_ to γ-TiAl and Ti6Al4V alloys was evaluated through the analysis of the results of the microstructural and chemical characterizations of the brazed interfaces.

OM and SEM images of the interface reveal that the brazing filler was effective in promoting the joining of Al_2_O_3_ to both γ-TiAl and Ti6Al4V alloys, after brazing at 980 °C with a dwelling stage of 30 min. Multilayered interfaces, apparently free of pores and cracks, were produced for both brazed systems of materials.

**Figure 1:**
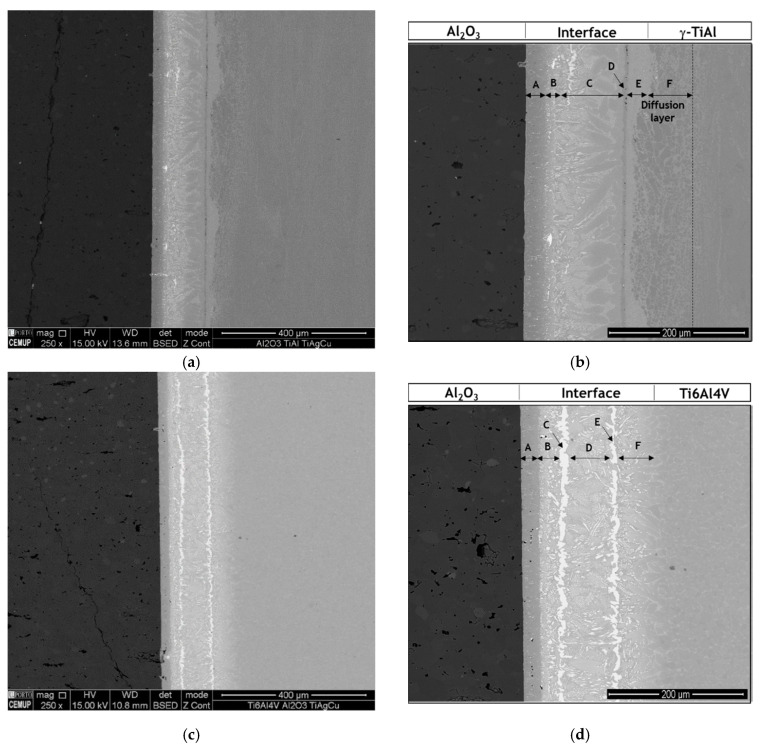
Scanning electron microscopy (SEM) images of the brazed interfaces of: (**a**,**b**) Al_2_O_3_/γ-TiAl alloy, and (**c**,**d**) Al_2_O_3_/Ti6Al4V joints, produced at 980 °C for 30 min.

Figure 1 shows SEM images of the interfaces of Al_2_O_3_/γ-TiAl alloy and of Al_2_O_3_/Ti6Al4V joints. By the observation of Figure 1, it is evident that the microstructure of the interface is strongly influenced by the type of Ti alloy used as base material.

The Al_2_O_3_/γ-TiAl alloy interface can be divided into five different layers: a layer close to Al_2_O_3_ (Layer A), a thin layer composed of a dark matrix with bright particles dispersed (Layer B), a central layer (Layer C), a dark thin layer (Layer D), a light-grey layer (Layer E) and a diffusion layer (Layer F).

The Al_2_O_3_/Ti6Al4V interface exhibits a more complex microstructure and can be divided into six distinct reaction layers: a layer close to Al_2_O_3_ (Layer A) mainly composed of a dark-grey phase, followed by a layer consisting of a mixture of grey and bright particles (Layer B), two thin layers (Layers C and E) each bordering the central layer (Layer D) and both essentially composed of a bright phase, and finally, a layer formed close to Ti6Al4V (Layer F) that presents a similar microstructure to Layer B, although slightly coarser.

**Figure 2:**
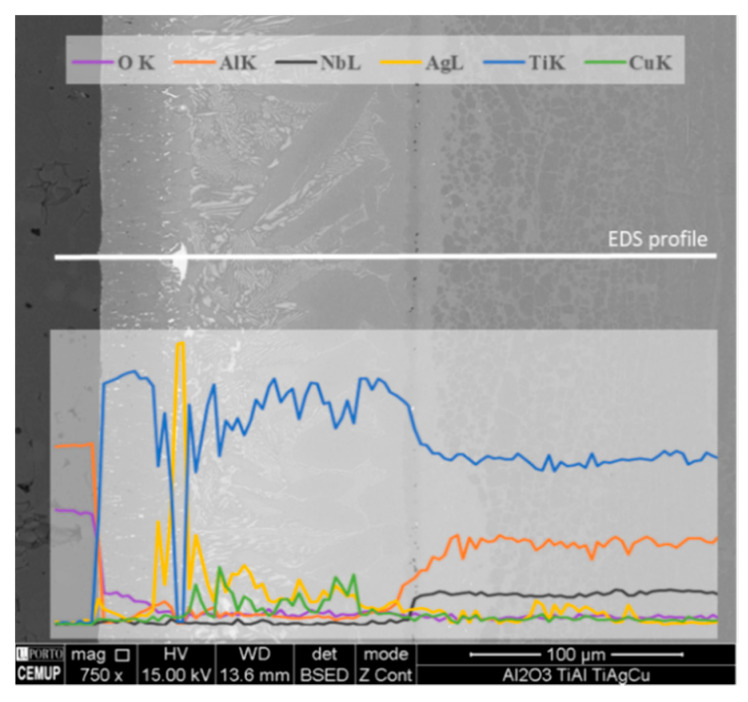
SEM image and energy dispersive X-ray spectroscopy (EDS) line profile on the interface of the brazed Al_2_O_3_/γ-TiAl alloy joint produced at 980 °C for 30 min.

An EDS line profile in the SEM image of the Al_2_O_3_/γ-TiAl alloy interface is presented in Figure 2, showing that the layer close to Al_2_O_3_ (Layer A) is Ti-rich. A small declining gradient of oxygen is also observed in this layer, which may depict the formation of oxide(s) phase(s). The line profile also indicates that the sporadic larger bright particles observed close to Al_2_O_3_ are Ag-rich. Although Ag and Cu are detected in greater amounts on the Al_2_O_3_ side, these elements are present throughout the entire interface and Ag is also detected in the diffusion layer of the γ-TiAl alloy, to an approximate depth of 70 µm from the interface.

In order to identify the possible phases constituting the reaction layers formed at the interfaces, EDS chemical analyses were performed in conjunction with SEM observations and combined with the information provided by the Ti-Al [32], Ti-Al-Ag [33,34], Ti-Ag-Cu [33], Ti-Al-V [35], Ti-Al-Nb [36] and Ti-Al-Cu [37] equilibrium phase diagrams.

**Figure 3:**
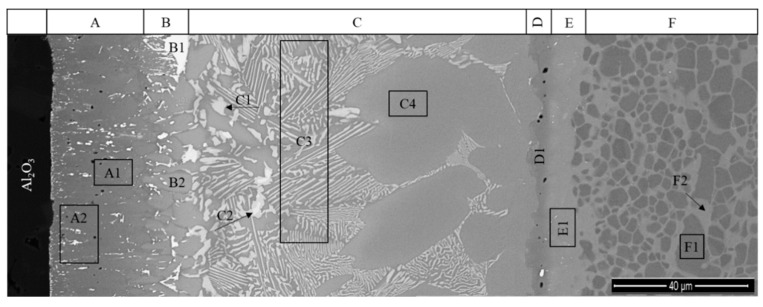
SEM image of Al_2_O_3_/γ-TiAl alloy interface with indication of the EDS analyzed zones in the reaction layers (**A**–**F**) of the interface.

**Figure 4:**
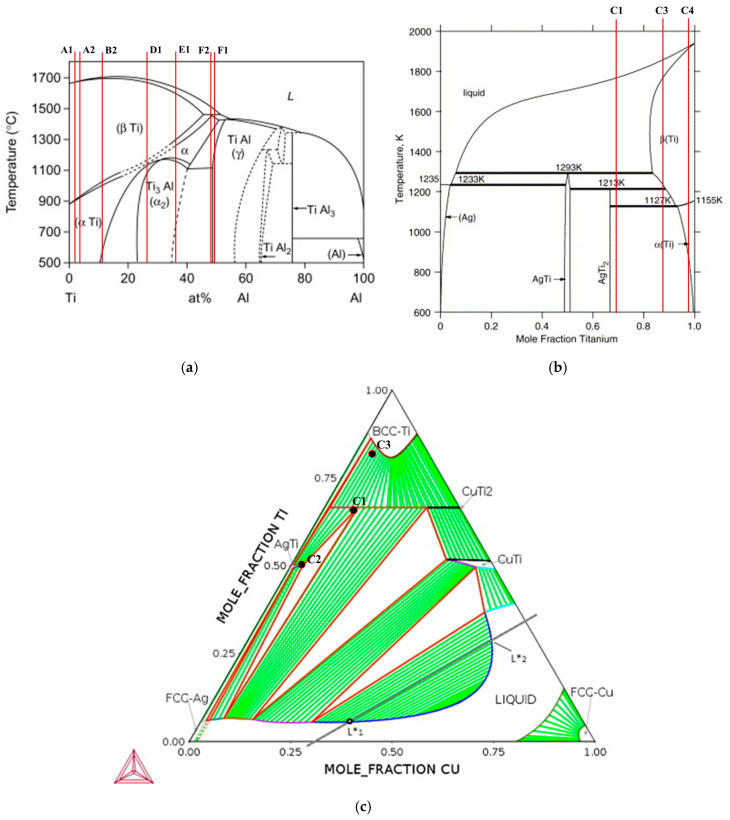
(**a**) Ti-Al [38], (**b**) Ti-Ag [39] and (**c**) Ti-Ag-Cu [40] phase diagrams with the EDS chemical composition of the zones marked in the SEM image of Al_2_O_3_/γ-TiAl alloy interface. Adapted with permission from [38,39,40]. Copyright 2005, 2015, 2018 Elsevier.

The Al_2_O_3_/γ-TiAl alloy multilayered interface can be observed in more detail in the SEM image presented in Figure 3. The EDS analysis result of each selected zone marked in Figure 3 is shown in Table 1. The Ti-Al [38], Ti-Ag [39] and Ti-Ag-Cu [40] phase diagrams with indication of the chemical compositions of the analyzed zones are presented in Figure 4, to explain the estimate of the possible nature of the main phases formed at the interface.

Layer A is essentially composed of Ti and consists of a grey matrix where bright particles are dispersed. The grey matrix (zone A1) should be α-Ti since it is composed of more than 98% Ti. Although the size of the white particles is smaller than the interaction volume of the EDS analysis, the results of zone A2 show an enrichment of Ag that is not observed in zone A1. Thus, it is reasonable to accept that the white particles may consist of a Ag-rich phase, which would result from the amount of Ag diffused from the filler foil having exceeded the low solubility limit of Ag in the α-Ti phase. It is also worthy of mentioning that the formation of a complex oxide layer at the vicinity of the Al_2_O_3_ was mentioned by some authors [27,29], when these base materials were joined using a commercial Ti-Ag-Cu brazing alloy. However, the formation of an oxide layer was not observed through the SEM/EDS characterization performed in the present study; this may be due to the fact that the oxide reaction layer is too thin to be detected by the techniques used in this investigation.

Layer B, which is located in the vicinity of Layer A, is mainly composed of α-Ti, mixed with a small amount of Ti-Ag intermetallics. Occasionally, white coarser particles (zone B1) consisting of (Ag) are observed in this layer, as illustrated in Figure 1, Figure 2 and Figure 3. The chemical composition of zone B2 lies on the (α-Ti + TiAg) two-phase field on the isothermal section of the Ti-Al-Ag phase diagram (see Figure 4). However, based on the observation of the SEM image, this zone must be composed of a single phase. Since the chemical composition obtained by EDS is rather close to the α-Ti field, it can be concluded that it should be mainly constituted of α-Ti. It should be noted that the light-grey particles observed in this layer present the same chemical composition as the related particles observed in Layer C, corresponding to zones C1 and C2; these zones should consist of Ti_2_(Ag,Cu) and TiAg, respectively, in both layers.

The central zone of the interface (Layer C), is mainly composed of α-Ti + Ti_2_(Ag,Cu) with a few dispersed TiAg particles (brighter particles). As it can be observed in Figure 3, Layer C is not uniform. In the vicinity of Layer B, Layer C is mainly composed of a mixture of α-Ti, Ti_2_(Ag,Cu) and TiAg, but near Layer D, the predominant phase is clearly α-Ti.

Layer D should be mainly composed of α_2_-Ti_3_Al and, along with Layer E, which is composed of a mixture of α_2_-Ti_3_Al + γ-TiAl, promote bridging to the γ-TiAl alloy. Layer F is a diffusion layer that extends into the γ-TiAl alloy and is essentially composed of γ-TiAl intermetallics. It should be noted that Ag is detected up to ~3% (in %) in this layer. In previous works [30,31], a similar diffusion layer was already observed at the interface of γ-TiAl alloy joints processed using the same Ag-Cu sputter-coated Ti brazing filler. The formation of this layer was explained based on the interdiffusion occurring between the γ-TiAl alloy and the brazing filler foil in the course of the joining thermal cycle. As a result of this process, the α_2_-Ti_3_Al phase from the (α_2_ + γ) lamellar grains of the duplex microstructure was transformed into γ-TiAl, originating from a diffusion zone essentially composed of γ-TiAl. Contrarily to the single-phase γ-TiAl grains of the duplex microstructure (zone F1), the γ-TiAl grains resulting from the transformed lamellar grains (zone F2) dissolved a small amount of Ag.

**Figure 5:**
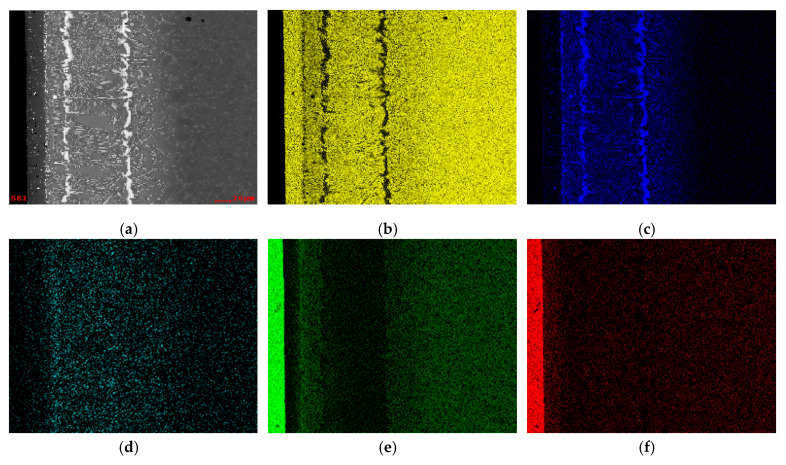
(**a**) SEM image and (**b**) Ti, (**c**) Ag, (**d**) Cu, (**e**) Al and (**f**) O EDS elemental maps of the brazed interface of Al_2_O_3_/Ti6Al4V joints produced at 980 °C for 30 min.

Regarding the Al_2_O_3_/Ti6Al4V brazed interface, EDS elemental distribution maps of Ti, Al, Ag, Cu and O are shown in Figure 5. Based on the Ti map, the layer close to Al_2_O_3_ is rich in Ti and, in fact, this element is present throughout the whole interface; this result is similar to what has already been mentioned for the interface with a γ-TiAl alloy as base material. Based on the Ag map, it can be seen that the brightest layers and particles observed at the center of the interface are rich in this element. In addition, in the layer closest to Al_2_O_3_ it is also possible to observe that the smallest bright particles are rich in Ag. The distribution map of Cu shows that this element is dispersed throughout the entire interface, but appears to be in more significant amounts near the ceramic. Contrary to the Al_2_O_3_/γ-TiAl interface, a diffusion layer is not detected on the Ti6Al4V base material.

**Figure 6:**
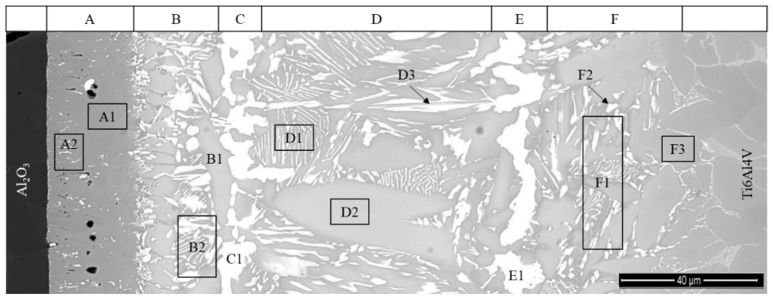
SEM image of the Al_2_O3/Ti6Al4V interface with marked EDS analysis zones in the reaction layers (**A**–**F**) of the interface.

Figure 6 shows the zones on the interface of the Al_2_O_3_/Ti6Al4V joint where the EDS analysis were performed. The chemical compositions of these zones are presented in Table 2.

Layer A is similar to the one observed close to Al_2_O_3_ at the Al_2_O_3_/γ-TiAl alloy interface and is composed of α-Ti and small Ag-rich particles. Layer B is composed of a mixture of α-Ti, Ti_2_(Ag,Cu), and TiAg. A noticeable feature of the Al_2_O_3_/Ti6Al4V interface, establishing a marked difference in comparison to the Al_2_O_3_/γ-TiAl alloy interface, is the formation of Layers C and E, which are nearly parallel to each base material surface and composed essentially of bright TiAg particles. Located between these layers, Layer D consists of a mixture of lamellar constituent (α-Ti + Ti_2_(Ag,Cu)), coarse α-Ti (zone D2) grains and bright TiAg particles (zone D3) that are generally elongated and roughly perpendicular to the base materials. Layer F is a reaction layer, mainly composed of α-Ti and Ti_2_(Ag,Cu) that promotes bonding to the Ti6Al4V alloy.

**Figure 7:**
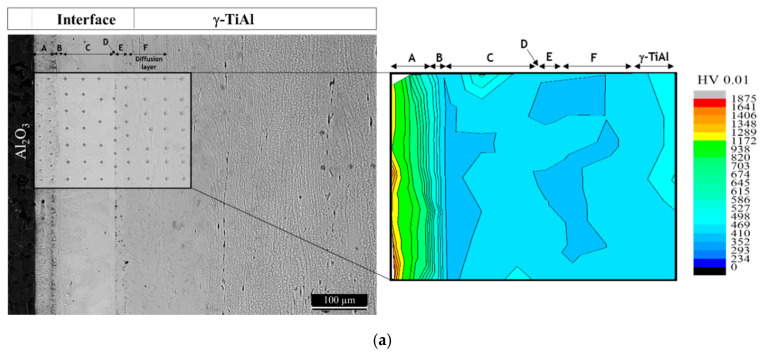

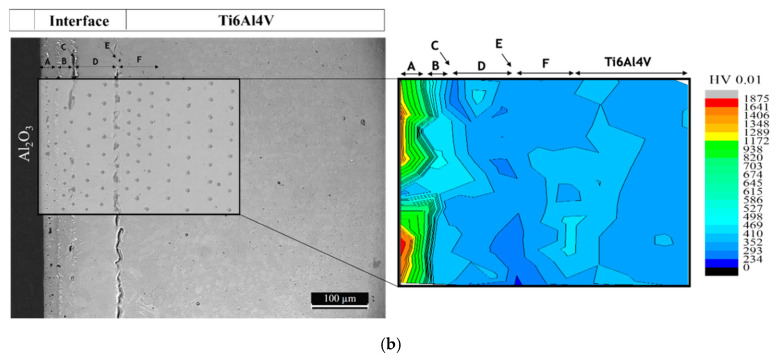
Optical microscopy (OM) image and hardness distribution maps of the (**a**) Al_2_O_3_/γ-TiAl alloy, and (**b**) Al_2_O_3_/Ti6Al4V brazed joints.

The hardness distribution maps of the brazed joints are presented in Figure 7. The maps show the exception of the region corresponding to Layer A; the hardness variation is rather smooth throughout both interfaces and similar to the corresponding Ti base alloy. However, in Layer A, the hardness is much higher than in the other regions of the interface, reaching 1872 HV0.01—close to Al_2_O_3_. In addition, Layer A presents a sharp hardness gradient, as it decreases to 857 HV HV0.01 near Layer B. The hardness values presented by Ti-rich Layer A indicate that it should be essentially composed of hard phases, instead of only α-Ti. As reported in several studies [28,29], the reaction between Al_2_O_3_ and the Ti/Ag-Cu brazing filler must have resulted in the formation of a reaction layer, possibly essentially consisting of hard Ti oxides and/or mixed Ti-Al oxides. These phases play a crucial role in the establishment of joining, as they will ensure chemical compatibility and bonding between the ceramic base material and the metallic phases formed at the interface. The interfacial hardness values of the regions in Layer A near Al_2_O_3_ are consistent with the formation of Ti oxides and/or mixed Ti-Al oxides.

The microhardness maps also show that the segregation of brittle particles towards a specific zone of the interface or extensive formation of soft phases was avoided, contrary to what is reported in other studies. For instance, the use of Ag-based fillers was reported to induce the formation of large (Ag) segregation zones [26,27,28,29], limiting the operating temperature of joints to around 350 to 400 °C. In contrast, such features were prevented in the present investigation. This indicates that the use of the novel Ag-Cu sputter-coated Ti brazing filler potentially avoids the need for eventual post-joining heat treatments to eliminate or minimize the undesirable segregated phases formed at the brazed interfaces.

**Figure 8:**
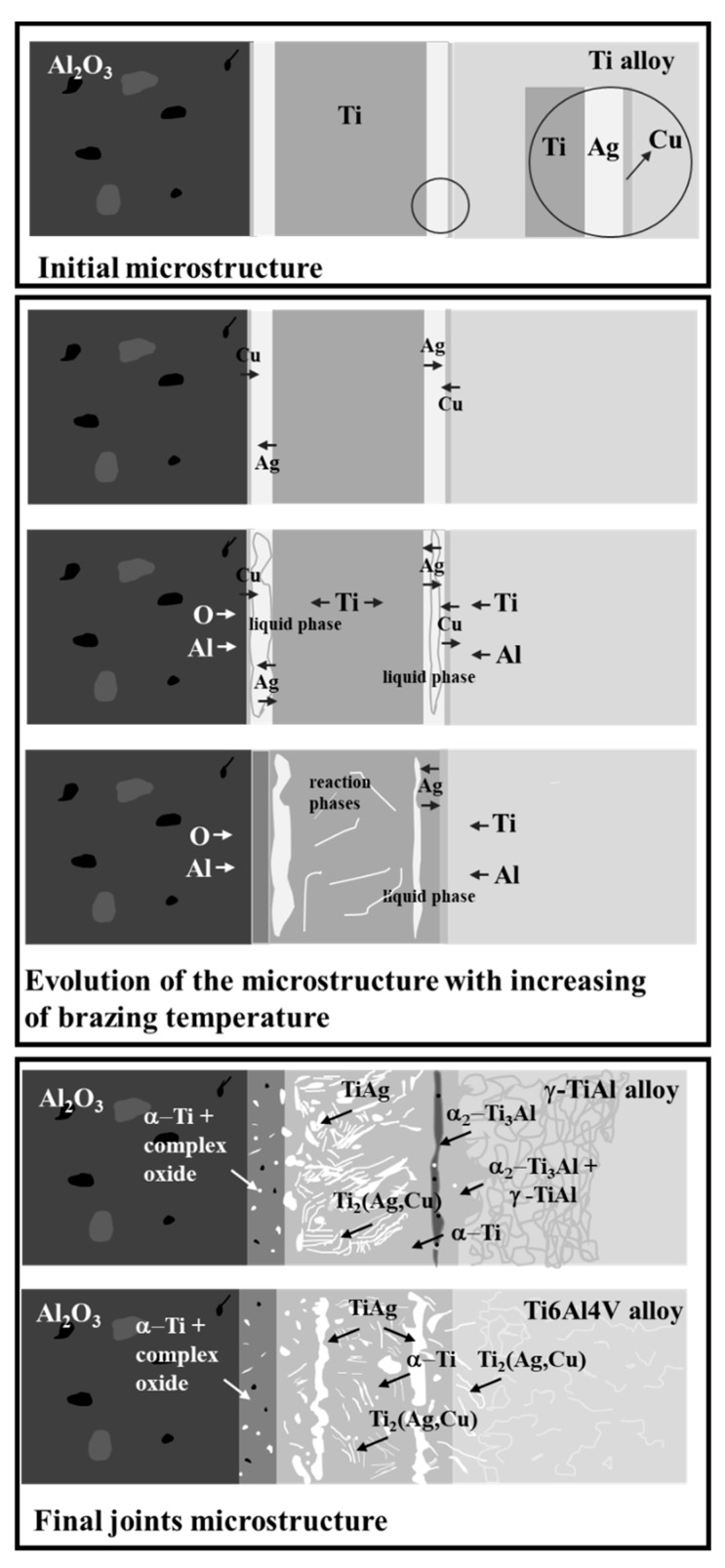
Schematic illustration of the mechanism for the evolution of microstructure in the interfaces obtained in this study.

Since the results are similar to those observed in previous studies [30,31] and despite the significant influence of the type of Ti alloy used as the base material on the microstructure of the interface, a common mechanism can be pointed out to explain the formation of the microstructure of the interface in the joining of Al_2_O_3_ to Ti alloys. Figure 8 shows a schematic illustration of a possible mechanism for the formation of the interfaces.

Based on the reaction between the Ag- and Cu-sputtered films observed for different brazing temperatures for similar γ-TiAl alloy joints [31], interdiffusion within the brazing filler originates from the formation of an Ag-Cu-rich liquid phase upon heating to the brazing temperature. As soon as it is formed, this liquid begins to dissolve the neighboring zones (reaching eventually the Ti sputter-coated foil and the base materials), until the melt solubility limits are exceeded. Thus, on the Ti alloy base material side of the interface, the liquid will incorporate mainly Ti and Al, while on the Al_2_O_3_ side, the liquid will be enriched in Ti, Al and O. As the solubility limits are exceeded, precipitates will consolidate into continuous layers, enabling bridging between the base materials and the filler. Near Al_2_O_3_, besides a layer composed of α-Ti and small Ag-rich particles, a thin Ti-Al-O oxide layer may ensure bonding between the base ceramic and the metallic braze. The Ag-rich particles are formed probably in part due to the fact that Ag diffusion into the Al_2_O_3_ is rather limited, since no Ag was detected by the EDS analysis in Al_2_O_3_, not even near the interface, in contrast to the γ-TiAl alloy. The joining of the γ-TiAl alloy is promoted by the (α_2_-Ti_3_Al + γ-TiAl) layer, while a layer composed of α-Ti and Ti_2_(Ag,Cu) ensures bonding to Ti6Al4V.

However, the Ag- and the Cu-sputtered films are not fully consumed during the liquid phase formation process. So, as interdiffusion is occurring throughout the forming interface, the remain Ag and Cu either dissolve in the base materials or react with Ti and Al, leading, in this case, to the formation of Ti-Ag-Cu intermetallics throughout the central zone of the interface. The extent of diffusion is, however, different depending on the Ti alloy base material. For example, the diffusivity of Ag in the γ-TiAl and α_2_-Ti_3_Al phases, which are composed of the base γ-TiAl alloy, appears to be higher than in the α-Ti and β-Ti phases which constitute the Ti6Al4V microstructure. This difference in the diffusivity may justify some of the differences between the interfaces obtained when brazing the two different Ti alloys. For instance, in the case of the γ-TiAl alloy interface, the reaction products formed near the base material present quite low Ag contents. However, a rather extensive diffusion layer containing Ag was detected and extended to about 70 µm into the base intermetallic alloy. In contrast, the volume fraction of Ag-rich intermetallics of reaction products formed near the base of Ti4Al6V is significant, but in this case, no diffusion layer was detected.

## 4. Conclusions

The joining of Al_2_O_3_ to γ-TiAl and to Ti6Al4V alloys was investigated, using Ag-Cu sputter-coated Ti foil as brazing filler. Brazing was carried out at 980 °C for 30 min in vacuum and sound multilayered interfaces were obtained for both systems of joined materials.

Bonding to Al_2_O_3_ was promoted by the formation of a Ti-rich layer, possibly consisting of a mixture of α-Ti and Ti oxides. Joining to the γ-TiAl alloy and to Ti6Al4V was ensured by a (α_2_-Ti_3_Al + γ-TiAl) layer and by a (α-Ti + Ti_2_(Ag,Cu)) layer, respectively. The central zones of the interfaces consisted mainly of α-Ti, Ti_2_(Ag,Cu) and TiAg.

The hardness throughout both interfaces is relatively homogeneous, and similar to the hardness of both base Ti alloys; exception made to the Ti-rich layer formed near Al_2_O_3_. This layer is the hardest region of both interfaces and exhibits a steep declining hardness gradient, with approximate hardness values of 1872 HV0.01 close to Al_2_O_3_ and 857 HV HV0.01 near Layer B.

The results obtained in this investigation indicate that the use of Ag-Cu sputter-coated Ti foil as brazing filler has significant advantages for joining Al_2_O_3_ to either γ-TiAl and Ti6Al4V alloys. Indeed, this filler promotes the formation of more homogeneous interfaces, with a significant reduction in either (Ag) and brittle intermetallic phases, in comparison to interfaces obtained using other Ti-Ag-Cu brazing alloys, or composite brazing fillers reported in the literature. Therefore, it could potentially avoid the need post-joining heat treatments to eliminate or minimize the undesirable phases formed at the interfaces.

## Figures and Tables

**Table 1:** EDS chemical composition (in %) of the zones of interest illustrated in Figure 3.

Zone	Al	Ag	Ti	Cu	Nb	Possible Main Phase(s)	Thickness (µm)
A1	1.1	-	98.9	-	-	α-Ti	35
A2	4.7	1.6	93.7	-	-	α-Ti	
B1	-	97.3	-	2.7	-	(Ag)	115
B2	11.3	7.6	78.7	2.4	-	α-Ti	
C1	2.4	21.3	65.7	10.6	-	Ti_2_(Ag,Cu)	
C2	-	48.7	48.1	3.1	-	TiAg	
C3	6.9	8.8	78.2	6.1	-	α-Ti + Ti_2_(Ag,Cu)	
C4	4.2	2.9	91.1	1.8	-	α-Ti	
D1	23.8	2.4	72.8	1.0	-	α_2_-Ti_3_Al	5
E1	31.1	2.4	59.5	1.3	5.7	α_2_-Ti_3_Al + γ-TiAl	30
F1	44.1	-	51.1	-	4.8	γ-TiAl	60–70
F2	40.9	2.9	51.5	-	4.7	γ-TiAl	
TiAl	42.3	-	52.0	-	5.7	γ-TiAl	-

**Table 2:** EDS chemical composition (in %) of the zones of interest illustrated in Figure 6.

Zone	Al	Ag	Ti	Cu	V	Possible Main Phase(s)	Thickness (µm)
A1	3.8	-	96.2	-	-	α-Ti	25
A2	2.8	1.4	95.8	-	-	α-Ti	
B1	10.6	7.6	79.1	2.7	-	α-Ti	120
B2	9.2	12.8	73.7	4.2		α-Ti + Ti_2_(Ag,Cu)+ TiAg	
C1	-	47.7	51.2	1.1	-	TiAg	
D1	2.1	8.3	85.2	4.4	-	α-Ti + Ti_2_(Ag,Cu)	
D2	1.9	5.9	91.1	1.1	-	α-Ti	
D3	-	49.7	47.1	3.1	-	TiAg	
E1	-	47.9	52.2	-	-	TiAg	
F1	6.7	6.9	82.9	1.4	2.1	α-Ti + Ti_2_(Ag,Cu)	55
F2	1.5	27.0	66.8	4.7	-	Ti_2_(Ag,Cu)	
F3	7.7	5.9	85.8	-	0.6	α-Ti	
Ti6Al4V	9.8	-	87.8	-	2.4	α-Ti + β-Ti	-
